# Data-driven personalized marketing strategy optimization based on user behavior modeling and predictive analytics: Sustainable market segmentation and targeting

**DOI:** 10.1371/journal.pone.0328151

**Published:** 2025-07-24

**Authors:** Bin Sun

**Affiliations:** School of Economics and Management, Changchun Finance College, Changchun, Jilin, China; Opole University of Technology: Politechnika Opolska, POLAND

## Abstract

Personalized recommendation remains a central challenge in modern marketing systems due to the complexity of user-product-query interactions. In this study, we propose a novel framework called DP-GCN (Deterministic Policy Graph Convolutional Network), which integrates multi-level Graph Convolutional Networks (GCNs) with Deep Deterministic Policy Gradient (DDPG) reinforcement learning to model heterogeneous information networks composed of users, products, and search queries. The proposed framework consists of three key components: (1) a graph-based embedding module to capture multi-relational structures; (2) a fusion layer that integrates dynamic and static features from users and items; and (3) a reinforcement learning layer that adaptively updates recommendation policies based on user feedback. We evaluate our model on several public benchmark datasets and a real-world dataset collected from a local e-commerce platform. Results demonstrate that DP-GCN consistently outperforms state-of-the-art baselines in AUC, Precision@K, and NDCG@K. The findings highlight the effectiveness of combining graph-based relational modeling with reinforcement learning for improving both the accuracy and adaptability of personalized recommendation systems.

## 1. Introduction

The relationship between data-driven user behavior predictive analytics and personalized marketing is close and interdependent, and this relationship has become increasingly important in recent years with the development of big data and machine learning technologies. User behavior predictive analytics uses a large amount of data collected from multiple channels to analyze users’ historical behaviors, preferences, social interactions, and consumption patterns through algorithmic models to predict future behavioral trends and potential needs [1]. Such predictions not only help companies understand the specific needs of users, but also provide the basis for implementing personalized marketing strategies. Personalized marketing, on the other hand, involves tailoring marketing messages and product recommendations to each user’s unique characteristics and predicted behaviors, thereby increasing the relevance and effectiveness of marketing. Modern personal marketing is a marketing strategy centered on digital technology, aimed at achieving precise and personalized brand promotion and customer interaction through social media, e-commerce, data analysis, and other means. It emphasizes the deep mining and insight of user data to understand audience needs, optimize customer journeys, and enhance brand stickiness. Social platforms such as WeChat, Tiktok and Xiaohongshu have become the main channels for personal marketing. Individuals can improve their exposure and conversion rate through content creation, community operation and live sales. In addition, with the help of artificial intelligence and automation tools, personal marketing has achieved efficient management of the entire process from customer acquisition to maintenance. At the same time, it also integrates content creativity and value output, building emotional connections with users through storytelling and establishing brand personality. Market segmentation is a foundational principle in marketing strategy, traditionally referring to the process of dividing a broad consumer market into distinct sub-groups based on shared characteristics. In the context of this study, segmentation is not based solely on static demographic data, but is dynamically inferred from users’ behavioral patterns, preferences, and query contexts. This behavioral segmentation enables more precise and sustainable targeting of marketing efforts. In short, modern personal marketing is not only a sales strategy, but also an art of establishing long-term relationships with consumers. In this way, companies are able to provide more relevant and effective services or products, increasing user satisfaction and loyalty, which in turn drives sales growth [2]. Personalized marketing is inherently linked to the broader concept of relationship marketing, which emphasizes building long-term, value-driven relationships with customers rather than focusing solely on single transactions. Personalized marketing can be considered a tactical approach within the relationship marketing paradigm, using data-driven personalization to reinforce trust, satisfaction, and loyalty. Without the foundation of relationship marketing, personalized efforts risk becoming short-term optimizations rather than sustainable customer engagement strategies. A personalized marketing strategy leverages user data and machine learning techniques to deliver highly relevant experiences, aiming to increase engagement, conversion rates, and long-term customer value.

With the advancement of technology, especially the application of artificial intelligence and machine learning, data-driven user behavior prediction and personalized marketing has evolved to a new level. Modern analytics tools and algorithms can analyze large-scale datasets in real time, not only predicting users’ immediate needs, but also adjusting marketing strategies in real time to adapt to changes in the market. Through machine learning, companies can build predictive models to accurately analyze a user’s purchase history and browsing behavior and predict their future behavior and preferences [3]. For example, algorithms such as Classification and Regression Trees (CART), Support Vector Machines (SVM), and Random Forests are able to identify the key factors that influence users’ decision-making and predict their likely purchasing behavior. Deep learning, especially Convolutional Neural Networks (CNN) and Recurrent Neural Networks (RNN), excel in handling complex user interaction data and time-series data, and are suitable for extracting sentiment and preferences from large amounts of user-generated content [4]. Reinforcement learning, on the other hand, optimizes marketing strategies through a continuous trial-and-error approach, enabling marketing campaigns to dynamically adapt to changes in user behavior. Through these methods, AI not only provides marketers with powerful data analysis capabilities, but also helps them implement decisions in a more scientific way to achieve precision marketing and optimize user experience, which ultimately promotes the long-term development and success of enterprises [5].

Therefore, in order to solve the core problem of how to accurately understand and predict the unique needs and behavioral patterns of each consumer faced by enterprises in the practice of personalized marketing. In this paper, we propose a personalized recommendation marketing model based on users’ historical data and related online shopping behaviors, with a view to enhancing consumers’ purchasing experience and strengthening their brand loyalty, thereby promoting sales growth and market competitiveness. The specific contributions of this paper are as follows:

(1) An intelligent recommendation framework DP-GCN based on reinforcement learning DDPG policy and GCN is proposed for the recommendation needs of personalized marketing and online shopping.
(2) Three-way feature fusion using separate user information, product information and user behavior improves the recommendation performance of DP-GCN, and its test results under public dataset are significantly better than traditional methods.
(3) After completing the tests under the public dataset, the model was tested for practical applications, and the results showed that its recommendation accuracy and recommendation satisfaction were higher than the traditional methods.

The rest of the paper is organized as follows: Section 2 introduces the related works about personalized recommendatio; The proposed framework DP-GCN is established in Section 3. Section 4 describes the experiment result and the practical test. Discussion is given in Section 5 and Conclusion is drawn at the end. Discussion is given in Section 5 and Conclusion is drawn at the end.

## 2. Related work

### 2.1. Traditional personalized recommendation

The research on the problem of personalized recommendation of commodities has roughly gone through three main development stages: at the beginning of the research, traditional machine learning methods were generally used to model user interests; subsequently, deep learning methods were started to be explored for personalized recommendation of commodities and a series of deep learning models were proposed with Embedding and MLP as the paradigm; and the current models of the newest level have formed a series of interest network-based deep learning models on the basis of their own On the basis of the proposed deep learning models, the concept of interest network is introduced to model the diversity of user interests, forming a series of deep learning models based on interest network [6]. Collaborative filtering recommendation is the most researched and popular strategy in personalized recommendation technology, which is mainly divided into two categories: user-based collaborative filtering and product-based collaborative filtering. This recommendation technology can mine and analyze complex unstructured objects and can mine knowledge patterns that differ greatly in content, which to some extent solves the shortcomings caused by its low degree of automation and the lack of richness of mining results [7]. Content-based recommendation is based on the user’s historical information, selecting items with a high degree of similarity to recommend to the user, but the features of the recommended objects have certain difficulties in extraction, such as movies, images, music, etc. There is no effective feature extraction method, and the system can not produce effective recommendations for new users, which affects the degree of satisfaction [8,9]. Therefore, with the development of data diversity and feature extraction diversity, machine learning and its corresponding improvement algorithms have been rapidly developed and applied.Embedding and MLP paradigm is one of the more widely used frameworks [10], which firstly maps all the items purchased by the user historically to multiple low-dimensional vectors through the Embedding layer, and then transforms the multiple low-dimensional vectors to the low-dimensional vectors in a certain way. Zhang et al. [11] proposed the FNN model by combining FM and MLP in tandem, and the FNN model uses the hidden vector obtained from FM pre-training. The hidden layer and its weights obtained from FM pre-training are used as the initial value of the first layer of the neural network, and the predicted results are finally outputted after the MLP network, so as to realize the corresponding personalized merchandise recommendation prediction and analysis task.

### 2.2. Deep learning approach personalized recommendation

Wang et al. [12] for the traditional collaborative filtering recommendation algorithm exists can not use the information outside the ratings and face the data sparsity problem, put forward a collaborative deep learning recommendation algorithm, through the self-encoder to extract the content information of the item, to alleviate the data sparsity problem that exists in the collaborative filtering recommender system; Wu et al. [13] improve the processing of the self-encoder, and put forward a collaborative denoising based auto encoder recommendation algorithm, to a certain extent, to improve the algorithm accuracy; Oord et al. [14] for the cold start problem in the recommendation algorithm, put forward a content-based deep music recommendation algorithm, through the deep convolutional neural network on the extraction of music features to alleviate the problem of cold start; Zheng et al. [15] put forward a use of the item review information of the depth of the recommendation algorithm, through the convolutional neural network to extract user review information, which potentially mitigates the scarcity problem and improves the quality of recommendation; Hidas et al. [16] will propose a session-based recurrent network recommendation algorithm, which views the user’s behavior of clicking on some items as a sequence, and mitigates the scarcity problem and the cold-start problem by training the recurrent neural network; Liu et al. [17] address the existing recommendation algorithm There is the problem of not being able to effectively deal with the time interval and spatial distance, proposed a location prediction algorithm based on recurrent neural network, through the extended recurrent neural network to solve the traditional algorithms in the modeling of successive time intervals and geographic distances of the existing problems; Wu et al. [18] proposed a recurrent recommendation algorithm, through the long and short-term memory neural network to extract the characteristics of the user’s behavior. The recommendation algorithm based on recurrent neural networks is a better method for user interest changes, but it also increases the complexity of the model.

Through the above research, it can be found that for shopping websites or other interest recommendation research, in the explosive growth of the amount of data and the form of data today, the traditional machine learning and statistical methods are difficult to meet the requirements. Deep learning-based recommendation algorithms do not have sufficient feature extraction on the input side of the data, and most of the feature fusion layer adopts a simple matrix decomposition form, which does not take into account the connection between the user and the item, and the existence of these problems greatly reduces the accuracy of the recommendation. Therefore, it is important to improve the feature input of the current method and use a more complex network structure that can realize multi-dimensional analysis in the network recommendation model to achieve more accurate recommendations.

## 3. Methodology

In this section, considering the market segmentation in personalized recommendation, a model-driven methodology combining Graph Convolutional Networks (GCN) and Deep Deterministic Policy Gradient (DDPG) to achieve personalized recommendation. GCN is used to extract multi-level structural features from a heterogeneous user-item-query graph, while DDPG enables dynamic policy optimization based on user feedback.

### 3.1. GCN

Graph Neural Network (GNN) is a type of neural network used to process data on graph structures. GCN is an important form of graph neural network which learns the representation of nodes by performing convolutional operations on graph structures [19]. In GCN, the new representation of each node is determined by a combination of its own features and the features of its neighboring nodes [20].The basic operation of GCN can be represented by the following equation:


$${\text{H}}^{\left(\text{l}+1\right)}=\sigma \left({\hat{\text{D}}}^{-\frac{1}{2}}\hat{\text{A}}{\hat{\text{D}}}^{-\frac{1}{2}}{\text{H}}^{\left(\text{l}\right)}{\text{W}}^{\left(\text{l}\right)}\right)$$ (1)

**Fig 1:**
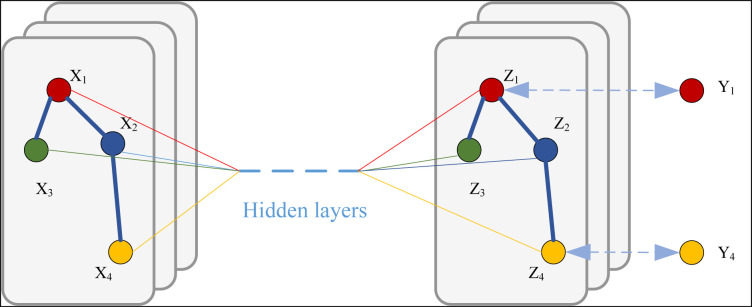
The framework for GCN.

$H^{\left(l\right)}$ is the node feature matrix of the first l node feature matrix of the layer, and$I_{N}$ is the unit matrix such that each node also considers its own features.$\hat{D}$ is the $\hat{A}$ the corresponding degree matrix.$W^{\left(l\right)}$ is the weight matrix of the first l weight matrix of the layer.σ is a nonlinear activation function, such as the ReLU function. In Graph Neural Networks (GCN), in addition to the basic graph convolution formulation, there are several other variants and extensions that can help to handle more complex scenarios and provide different perspectives to understand graph data. Its overall graph convolution process and the corresponding outputs are shown in Fig 1:

The aggregation function (i.e., how to merge the information of a node and its neighbors) is a key change point in the various variants of GCN, and this aggregation method places more emphasis on the capture of the most salient features among neighbors. The main ways of aggregation include weighted average aggregation and maximum pooling aggregation, and the process of both is shown in Eqs. (2) &(3):


$${\text{h}}_{\text{i}}^{\left(\text{l}+1\right)}\text{=}\sigma \left({\text{W}}^{\left(\text{l}\right)}\cdot \sum {\mathrm{\backslash nolimits}}_{\text{j}\in 𝒩(\text{i})\cup \{\text{i\textbackslash{}}}\}\;\;\frac{1}{{\text{c}}_{\text{ij}}}{\text{h}}_{\text{j}}^{(\text{l)}}\right)$$ (2)

$${\text{h}}_{\text{i}}^{\left(l\;+1\right)}=\sigma \left({\text{W}}^{\left(\text{l}\right)}\cdot \max_{\text{j}\in 𝒩(\text{i})}\;\text{ReLU}\left({\text{W}}_{\text{pool\textasciitilde{}}}{\text{h}}_{\text{j}}^{\left(\text{l}\right)}+\text{b}\right)\right)$$ (3)

where $c_{ij}$ is a normalization constant such as ${\hat{D}}_{ii}$, which is a simplified aggregation formula focusing on equal-weight neighbor feature aggregation. This type of aggregation method puts more emphasis on capturing the most salient features among neighbors. For a typical web-based product recommendation, I can abstract it into the following structure and make assumptions:

Given the set <Y,S,C,W,A,B>, where $Y=\left\{y_{I},\ldots ,y_{p}\right\}$ denotes the p the set of users, the set of $S=\left\{s_{l},\ldots ,s_{q}\right\}$ denotes the set of q the set of products, the set of $C=\left\{c_{l},\ldots ,c_{r}\right\}$ is the set of r the set of queries, the set of $W=\left\{w_{l},\ldots ,w_{n}\right\}$ is the set of n a collection of product vocabulary terms, the A represents the attributes associated with an object, the B represents the interaction behavior between different types of objects.

The user’s regular operation is to query c∈C or product s∈S consisting of several terms w∈W consisting of several terms. The purpose of personalized recommendations is to suggest to the user y∈Y suggest the most relevant intent (i.e., query) q∈Q.For a user performing an interest-click search, we leverage the adapted Graph Convolutional Network (GCN) framework to predict and suggest queries that best match the user’s intent. In this context, the relevant user preferences are computed by integrating information from multiple sources, denoted as A and B, which may include historical interaction data, item attributes, user behavior patterns, or contextual signals. The GCN framework enables the effective aggregation and propagation of these heterogeneous features across the graph, capturing both direct and indirect relationships. In practice, the node embeddings for users, queries, and items are updated iteratively through type-specific graph convolutional layers. For each user y, the features propagated from related nodes (e.g., past clicks or similar users) are combined to refine the user’s current interest profile. Similarly, candidate queries are enriched with contextual information gathered from item attributes or other users with similar preferences. Once the embeddings are updated, the system computes relevance scores for multiple potential queries by matching them against the user’s dynamic interest profile. These scores reflect how closely each query aligns with the user’s inferred intent. Finally, the query with the highest relevance score is suggested to the user as the most likely expression of their intent, providing a seamless and personalized search experience.

### 3.2. DDPG

Reinforcement Learning (RL) is a subfield of machine learning that deals with the problem of an intelligent agent learning how to make optimal decisions in an environment. In this process, the agent learns by observing the state of the environment, performing actions, and receiving rewards. The goal is to maximize the cumulative reward [21]. Deep Deterministic Policy Gradient (DDPG) is an algorithm that combines deep learning and reinforcement learning and is particularly suited for problems in continuous action spaces.DDPG is an Actor-Critic architecture that combines the Deterministic Policy Gradient (DPG) and Deep Q Networks (DQN) advantages of the DDPG. Its main feature is the use of two networks, Actor for determining actions and Critic for evaluating the value of state-action pairs. Unlike common policy gradient methods, the Actor in DDPG directly outputs the optimal action, not the action probability distribution [22].

In the experience playback part of reinforcement learning, the model stores previous transitions (states, actions, rewards, new states) and samples them randomly to reduce sample correlation. In the final target network, in order to stabilize the learning, DDPG uses the target network technique, i.e., there is a set of slowly updating networks to generate stable target values. For DDPG, its main action and state evaluation update mainly consists of two steps, i.e., Critic’s update and Actor’s update, which are shown in Equation (4) and Equation (5):


$$\text{L}=\frac{1}{\text{N}}\sum {\mathrm{\backslash nolimits}}_{\text{i}}\;{\left(\text{Q}\left({\text{s}}_{\text{i}},{\text{a}}_{\text{i}}|\theta^{\text{Q}}\right)-\left({\text{r}}_{\text{i}}+\gamma {\text{Q}}^{\prime }\left({\text{s}}_{\text{i}+1},\mu^{\prime }\left({\text{s}}_{\text{i}+1}|\theta^{\mu^{\prime }}\right)|\theta^{{\text{Q}}^{\prime }}\right)\right)\right)}^{2}$$ (4)

$${\;{\;\nabla_{\theta^{\mu }}\text{J }\approx \frac{1}{\text{N}}\sum_{\text{i}}\;\;\nabla_{\text{a}}\text{Q}\left(\text{s},\text{a}|\theta^{\text{Q}}\right)|}_{\text{s }={\text{s}}_{\text{i}},\text{a}=\mu \left({\text{s}}_{\text{i}}\right)}\nabla_{\theta^{\mu }}\mu \left(\text{s}|\theta^{\mu }\right)|}_{{\text{s}}_{\text{i}}}$$ (5)

where $\theta^{Q}$ are the parameters of Critic, the $Q^{\prime }$ and $\mu^{\prime }$ are the networks of the target Critic and the target Actor, and γ is the discount factor.The Actor network maximizes the Q value given by Critic by gradient ascent. In DDPG, the intelligent body selects actions by current strategy and exploration noise, observes the new state and rewards after execution, and stores these experiences into the experience playback pool. During training, a random batch of experiences is drawn from the experience playback pool, and the Critic network is first updated, which learns by minimizing the squared error between the predicted Q-value and the target Q-value (computed using the target Actor and the target Critic network). Then, the Actor network is updated using Critic’s output so that it outputs actions that maximize the Q value given by Critic. In addition, through soft updates, the parameters of the target network are slowly adjusted to ensure training stability. This process is looped until the performance criteria of the algorithm are met. In my marketing problem, the Deep Deterministic Policy Gradient (DDPG) algorithm offers a powerful solution by addressing the challenges of sequential decision-making in dynamic environments. Specifically, our goal is to optimize personalized marketing strategies, such as recommending products, setting discounts, or selecting targeted advertisements, based on users’ real-time interactions and evolving preferences. DDPG, as a model-free reinforcement learning algorithm, is well-suited for continuous action spaces, enabling fine-tuned marketing decisions. In our framework, the agent represents the marketing system, interacting with users through recommendations or offers (actions) and receiving feedback through metrics like click-through rates, conversions, or engagement (rewards). The state space includes contextual factors such as user profiles, browsing history, and time-sensitive behaviors, which are continuously updated. The algorithm leverages an actor-critic structure, where the actor generates optimal marketing actions, and the critic evaluates their effectiveness. Through exploration and exploitation, DDPG learns to maximize long-term rewards, adapting its strategy over time to offer increasingly relevant marketing content. This approach allows for personalized, real-time optimization, ensuring that marketing efforts remain both effective and user-centric, enhancing engagement and conversion rates across diverse audiences and scenarios.

### 3.3. DP-GCN

**Fig 2:**
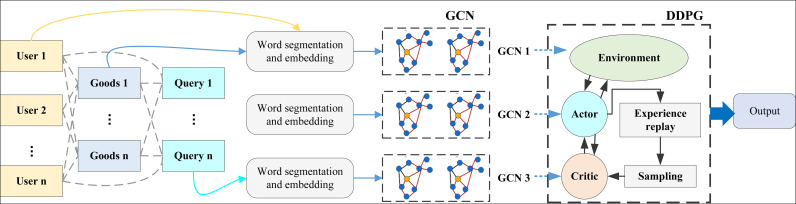
The framework for DP-GCN. This study adopts a three-object heterogeneous information network comprising users, items, and queries as input to model and optimize the complex relationships involved in personalized recommendations. First, I apply tokenization to the input data, breaking down the textual information of users, items, and queries into word units. We then generate initial embeddings for users, items, and queries using pre-trained vectors or random initialization with the same dimensionality. These embeddings capture the basic semantic information of each entity, laying the foundation for subsequent relationship modeling.

After completing the introduction of graph neural networks and reinforcement learning DDPG strategies, we built the DP-GCN framework that fuses multilevel GCN and reinforcement learning DDPG strategies based on the characteristics of data features and graph networks to carry out a multilevel fusion framework based on user behavioral features, commodity features, and query features, and the overall structure of the framework is shown in Fig 2:

Next, I employ a GCN (Graph Convolutional Network) model to aggregate the neighborhood information for each node. Different types of nodes (users, items, and queries) interact with their neighbors along specific paths within the graph, resulting in updated embeddings that encapsulate both their own attributes and the relational information from connected nodes. This process captures the complex relationships among users, items, and queries.

In the feature fusion layer, I incorporate necessary static features (such as user age, gender, and preferences) and combine them with the initial embeddings, feeding them into a multi-layer neural network for further processing, enhancing the model’s expressive power. In the decision layer, I fuse the dynamic embeddings of users, items, and queries with static features, achieving feature modeling for the three objects through three parallel GCNs: User modeling: Capturing user behavior and preferences.Item modeling: Analyzing item attributes and popularity.Query modeling: Understanding the alignment between queries and user needs.

During the decision-level fusion, I introduce the DDPG strategy to enable dynamic interactions between data and the environment. By leveraging reinforcement learning, the model continuously adjusts the recommendation strategy based on user behavior feedback, updating states to enhance recommendation accuracy and real-time performance. Finally, in the feature fusion stage, we integrate user static features, item embeddings, and query embeddings into a unified representation. These embeddings are fed into a multi-layer neural network to generate prediction results, estimating the probability of a user clicking on a specific query. The overall architecture achieves multi-source feature fusion based on graph neural networks and dynamic optimization through reinforcement learning, providing a more accurate and efficient solution for personalized recommendation systems.

The model output of this paper is shown in Equation (6):


$$\text{prediction = sigmoid}\left(f\left(Y_{i}⊕S_{i}⊕S_{ij}\right)\right)$$ (6)

where f(·) is an output layer with only one output, sigmoid (·) is the activation function, and ⊕ is the embedding crosstalk operation.The loss function used in DP-GCN is the cross-entropy loss function, which is calculated as shown in Equation (7):


$$L=\sum {\mathrm{\backslash nolimits}}_{i\in y\cup y^{-}}\;\left[y^{y}\text{log}{\left(y^{y}\right)}^{\prime }+\left(1-y^{y}\right)\text{log}\left(1-{\left(y^{y}\right)}^{\prime }\right)\right]$$ (7)

where $\left(y^{y}\right)$ is the label of the instance (i.e., 1 or 0)),y and $y^{-}$ are the set of positive and negative instances, respectively.

## 4. Experiment result and analysis

### 4.1. Experiment setup and model training

For the analysis of personalized marketing and market segmentation, its main goal is to analyze the corresponding interest characteristics of users by mining their historical data and behavioral data generated during the shopping process, so as to realize personalized marketing and improve sales and efficiency. Therefore, for the model, it needs to be analyzed and tested by shopping related historical data to realize its further application. The experimental environment used in this paper is shown in Table 1:

**Table 1:** The experiment environment.

Item	Specifications
CPU	Intel i5-8300
RAM	32G
GPUs	GTX 2080
Language	Python
Framework	Tensor flow

After completing the establishment of the experimental environment this paper confirms the datasets used, taking into account the characteristics of the data in the actual application this paper chooses the open source datasets on Amazon, and selects the Electronics (https://doi.org/10.5281/zenodo.3986936) and Books (https://doi.org/10.5281/zenodo.7555256) datasets [23]. Selecting the Electronics and Books datasets from Amazon’s open source dataset has significant advantages. Firstly, these two datasets cover a wide range of user groups and consumer behaviors. The Electronics dataset involves diverse electronic products such as mobile phones, computers, etc., reflecting highly active and frequent purchasing decisions; The Books dataset focuses on cultural and educational needs, reflecting long-term preferences and user interests different from fast-moving consumer goods. Secondly, both datasets provide rich user comments, ratings, and metadata, supporting research on sentiment analysis, recommendation systems, and user behavior prediction. By combining these two types of data, we can explore the differences in cross domain user behavior patterns and personalized recommendations. In addition, their data scale is large and highly structured, making them suitable for training and testing deep learning models, helping to develop efficient and accurate marketing strategies and recommendation systems. Choosing these two datasets enables the study to cover a wide range of consumer categories while also analyzing users’ deep level preferences. The basic information of the two types of datasets is shown in Table 2:

**Table 2:** The specific information for the employed dataset.

Name	User number	Goods number	Goods type	Total orders
Electronics	190000+	63,000+	800+	1700000+
Books	600000+	360000+	1600+	8900000+

The comparison of the database data in Table 2 shows that the total number of users and the total number of products in the Books dataset are significantly more compared to the Electronics dataset. Therefore, I can consider the former is a small dataset and the latter is a large dataset, which makes the model results more convincing by comparing the datasets of different sizes. In addition, for the above dataset, the data of each item contains the characteristics of product ID, product category, user’s history of shopping products, user’s history of shopping type and other fields.

The AUC curve and the RelaImpr metrics are used in this paper for comparison.The AUC (Area Under the Curve) curve is commonly used to evaluate the performance of classification models, especially in binary classification problems.The AUC curve is related to the ROC (Receiver Operating Characteristic) curve.The ROC curve describes the performance of the model at different thresholds by comparing the true case rate with the false positive case rate. curve describes the performance of the model at different thresholds by comparing the true case rate with the false positive case rate. The AUC, on the other hand, is the area under the ROC curve and provides a single value to measure the overall classification performance of the model.The RelaImpr metric is calculated as shown in (8):


$$\text{RelaImpr}\;=\left(\frac{\text{\textasciitilde{}\textbackslash{}text\{AUC}\;(\;\text{measured model}\;)-0.5}{\}}\text{AUC}(\;\text{base\textbackslash{} model}\;)-0.5-1\right)\times 100\;\%$$ (8)

During the model comparison I have chosen the more basic models including Embedding&MLP [10], Wide&Deep [24], PNN [25] and DIN [26] models. In addition to this we also compare a single GCN, with a D-GCN method that does not use feature fusion. The hyperparameter setting of the DP-GCN is illustrated in Table 3.

**Table 3:** DP-GCN Model Hyperparameter Settings.

Setting	Value
Optimizer	Adam
Batch Size	512
Number of Epochs	50
Learning Rate	1e-4
Discount Factor (γ)	0.99
Soft Update (τ)	0.005

During data preprocessing, I normalized all textual inputs (user queries, item descriptions, etc.) by lowercasing, removing stop-words, and applying basic stemming. Query disambiguation was conducted using TF-IDF-based similarity clustering and product category mapping, to resolve semantically similar but lexically different queries. Embedding vectors were uniformly set to 128 dimensions: pretrained Word2Vec embeddings were used for textual components, while numerical or categorical attributes were initialized randomly. To address the cold-start user problem, we utilized static user features and adopted a k-nearest neighbor strategy in the embedding space to generate initial user representations, which were refined during training.

### 4.2. Model comparison and result analysis

**Fig 3:**
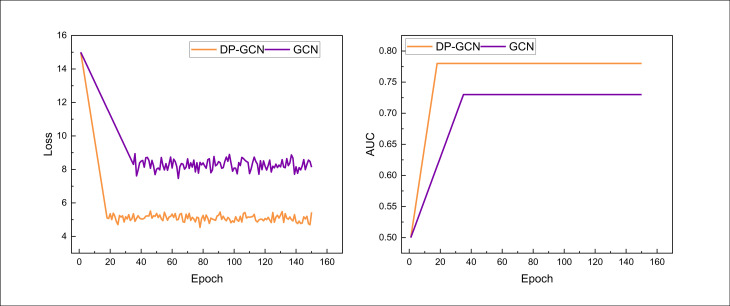
Training process and AUC value on the Electronics dataset.

**Fig 4:**
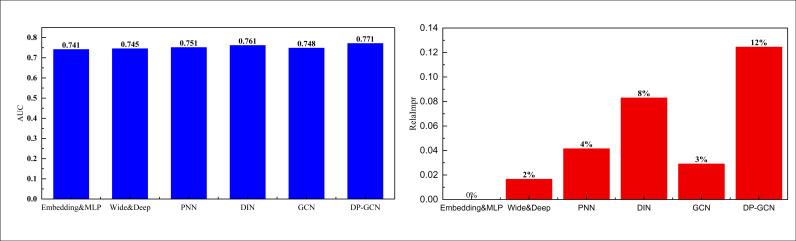
The comparison result on the Electronics dataset.

After determining the dataset and related data for model training, the public dataset was utilized for model training. This paper focuses on the analysis of the personalized marketing intelligence domain, so in the process of model training, the essence of the model is a classification and recognition problem based on multi-source data, and accurately identifies the most interesting class of products from a large number of candidate products. Fig 3 gives the loss function changes and the corresponding AUC changes during the model training process:

As can be seen from the training loss variation on the smaller dataset Electronics, through the improved dynamic interaction performance of reinforcement learning, the loss function is able to converge faster and remain stable, and the final AUC value is also maintained at a high level. Therefore, we compared the AUC values of the related models involved, and used the Embedding & MLP model, which has a long history of mediocre performance, as the corresponding base model for the performance calculation of the improved metrics. The AUC values and boosting metrics of the corresponding compared models are given in the two bar charts in Fig 4.

In Fig 4, I can see that the AUC of the proposed model on the Electronics dataset reaches 0.771, which is significantly better than traditional methods such as PNN. This performance improvement indicates that my model is more accurate and efficient in capturing user interests and behavior patterns. In addition, for the method using a single GCN, although its initial framework performance is slightly lower than some basic methods, the overall performance has been significantly improved by introducing DDPG for reinforcement learning training, from 0.748 to 0.771. This performance improvement is of great significance in personalized marketing scenarios. A higher AUC value indicates that the model performs more accurately in distinguishing whether users are interested in the recommended items, which directly improves the accuracy of personalized recommendations and user satisfaction. With the optimization capability of DDPG reinforcement learning, the model can dynamically adapt to changes in user behavior, continuously adjust recommendation strategies through interaction, and achieve real-time personalized marketing. With the improvement of model performance, marketing systems can more effectively increase click through rates and conversion rates, provide users with products and services that better match their interests, while avoiding unnecessary recommendation noise, enhancing user experience and brand stickiness.

As shown in Table 4, DP-GCN achieves the best performance across all ranking metrics, including Precision@5, Precision@10, NDCG@5, and NDCG@10. Compared to baseline models such as DIN and GCN, DP-GCN yields noticeable improvements, particularly in top-5 recommendation accuracy. These results indicate that the proposed model not only enhances overall prediction quality (as reflected in AUC) but also significantly improves the ranking relevance of recommended items, which is critical for practical recommendation scenarios.

**Table 4:** Precision@K and NDCG@K (K = 5, 10) Comparison on the Electronics dataset.

Model	Precision@5	Precision@10	NDCG@5	NDCG@10
Embedding&MLP	0.342	0.298	0.384	0.355
Wide&Deep	0.351	0.305	0.390	0.360
PNN	0.360	0.312	0.397	0.368
DIN	0.372	0.325	0.408	0.378
GCN	0.358	0.315	0.402	0.371
**DP-GCN (Ours)**	**0.401**	**0.349**	**0.438**	**0.399**

**Fig 5:**
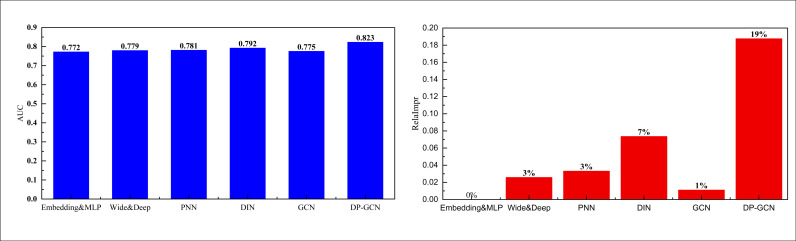
The comparison result on the Books dataset.

The results on the Books dataset are shown in Fig 5:

In the results of the Books dataset shown in Fig 5, our DP-GCN method demonstrated excellent performance, with an AUC value exceeding 0.8, significantly better than other comparison models. This result indicates that DP-GCN can more accurately capture users’ interests and preferences in the field of book consumption, thereby improving recommendation effectiveness. At the same time, the performance improvement of the model on the RelaImpr metric is also outstanding, reaching nearly 20%, indicating that its advantages in personalized recommendation are more obvious. This performance improvement is crucial for personalized recommendation marketing. Firstly, a higher AUC means that the model has stronger discriminative ability in predicting users’ interest in recommended books, which can reduce the frequency of irrelevant recommendations and improve user satisfaction. Secondly, the significant improvement in RelaImpr metrics indicates that our model not only enhances click through rates and conversion rates, but also outperforms the basic model in practical applications, providing users with more targeted marketing content. In order to comprehensively evaluate the practical application value of the model, we also analyzed the running time of the model on the test set. Through this analysis, we can verify whether the model has sufficient computational efficiency to support real-time personalized recommendation while improving performance. Overall, the efficient performance and significant effects of DP-GCN fully meet the needs of personalized marketing, providing strong technical support for the platform to enhance user experience and commercial revenue.

**Fig 6:**
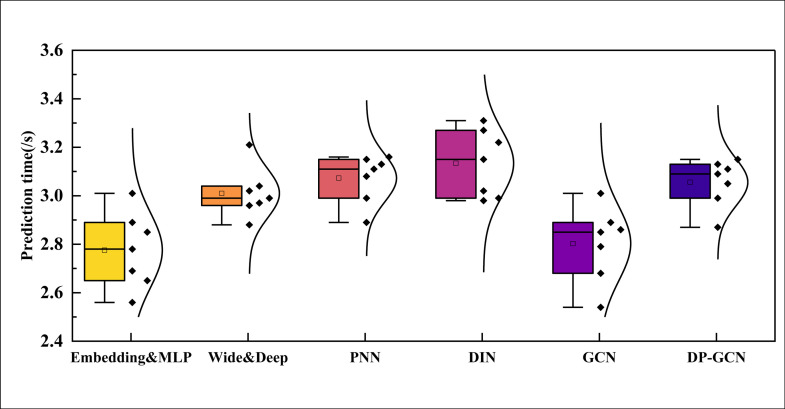
The prediction time comparison on Books dataset.

On Books dataset, as presented in Table 5, DP-GCN again outperforms all baseline models with notable margins in both Precision@K and NDCG@K. The improvements are particularly significant at top-5 rankings, where DP-GCN reaches a Precision@5 of 0.421 and NDCG@5 of 0.453. These results align with the AUC and relative gain trends shown in Fig 5, further validating the effectiveness and generalizability of our model across datasets. In the process of dividing the test set of data, we chose multiple random seeds to realize data division, and the box plots of the running time under different test sets are shown in Fig 6:

**Table 5:** Precision@K and NDCG@K (K = 5, 10) Results on Books dataset.

Model	Precision@5	Precision@10	NDCG@5	NDCG@10
Embedding&MLP	0.338	0.294	0.376	0.342
Wide&Deep	0.345	0.301	0.383	0.350
PNN	0.353	0.308	0.390	0.357
DIN	0.367	0.322	0.404	0.370
GCN	0.349	0.310	0.395	0.360
**DP-GCN (Ours)**	**0.421**	**0.367**	**0.453**	**0.414**

In Fig 6, I can see that the method proposed in this paper does not have a fixed advantage in computing time, and its overall average time is above and below 3s, with a higher rate than that of methods such as the base model and the PNN, but the difference in the overall level is all less than 1s, which indicates that the method proposed in this paper has a certain degree of efficiency in improving the efficiency of the model recommendation, and realizes a very good effect and performance. Although the time efficiency improvement is not the main advantage of the framework, the response time of 3 seconds is fast enough in real-world applications to meet the needs of most real-time recommender systems. This means that companies can maintain high levels of recommendation accuracy and personalization while still ensuring that the user experience doesn’t suffer due to long wait times.

In a world where user experience is increasingly emphasized, a responsive recommendation system can significantly increase user satisfaction and engagement, which in turn helps to improve user conversion and retention rates. Second, for market segmentation and personalized marketing, even minor efficiency gains can be extremely valuable. In a competitive market environment, the ability to quickly and accurately recommendation products that match users’ interests and needs will have a direct impact on a company’s revenue and market share. the DP-GCN framework provides accurate personalized content through in-depth analysis of user data and behavioral patterns, enabling companies to more effectively reach their target users, and optimize the effectiveness of advertising and promotional resources. Finally, for intelligent recommendation of online goods, the framework provides a highly personalized and accurate recommendation method by integrating user, product and query data. This approach not only improves the relevance and attractiveness of recommendations, but is also suitable for application in e-commerce platform environments that require fast decision-making due to its relatively fast response time. A fast and accurate recommendation system can better satisfy users’ immediate shopping needs and increase their willingness to buy, thus driving sales growth and customer loyalty.

### 4.3. Model application and practical test

**Fig 7:**
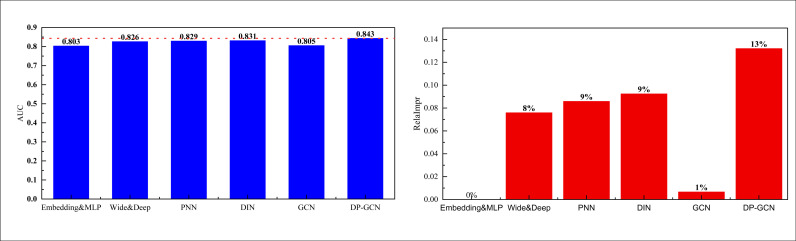
The comparison result on the self-established dataset.

**Fig 8:**
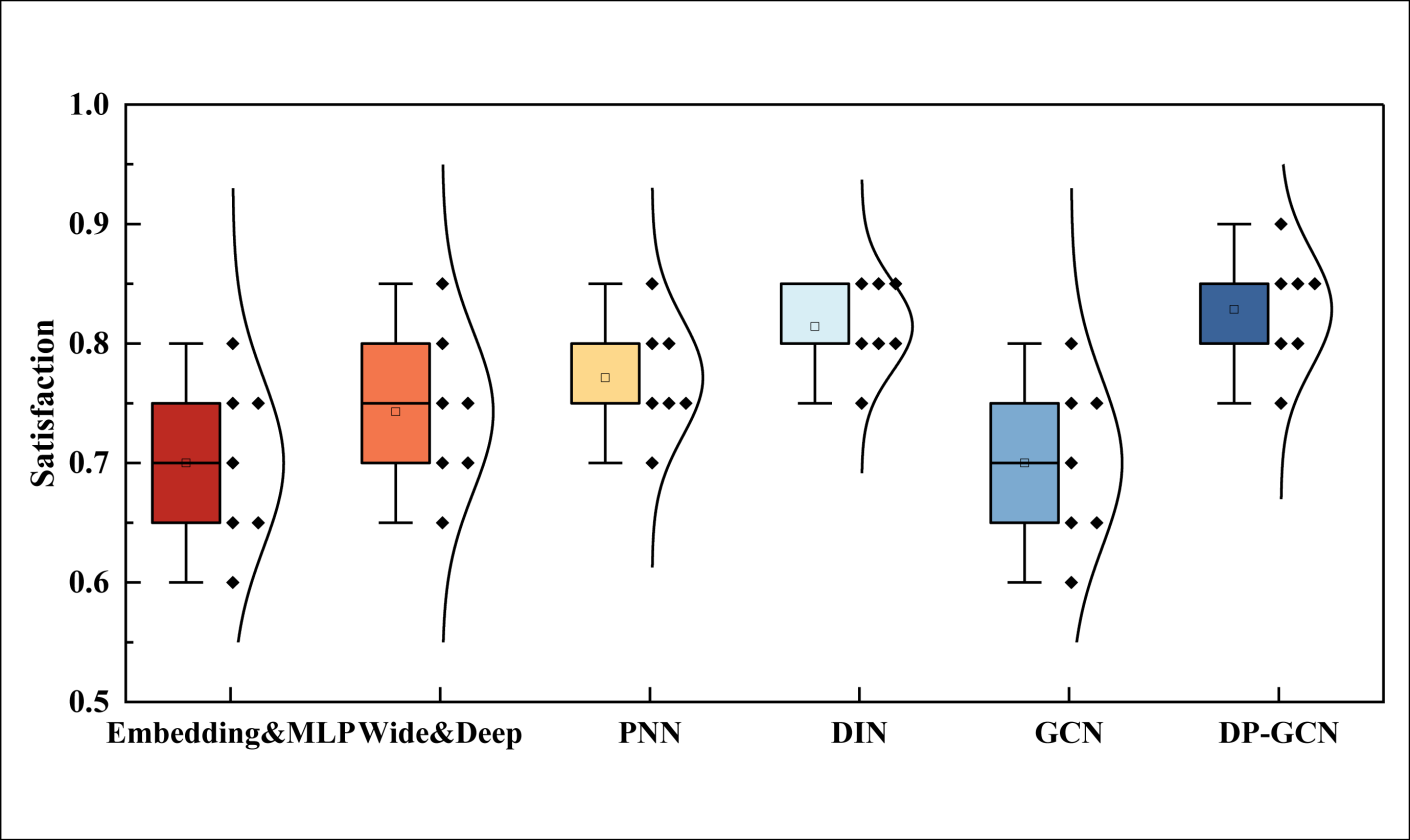
The user satisfaction comparison.

After completing the testing and analysis of the model on the public dataset, we selected the recommendation efficiency of a product in the region and compared it with the user’s final purchased product according to the characteristics of the product marketing in the region and the characteristics of the website data, and the results were obtained as shown in Fig 7:

To evaluate the model in a real-world context, I constructed a self-built dataset focusing on a local product category (e.g., small household appliances). A total of 500 users were selected based on recent activity over a three-month period, ensuring diversity in demographics and browsing patterns. The product set included over 300 distinct SKUs spanning 15 subcategories, providing a rich recommendation space. We labeled the data by comparing each user’s actual purchased item with the top-N recommendation results. If the purchased item appeared in the top-5 predictions, it was counted as a successful recommendation. In addition, we collected optional satisfaction feedback from 83 users using a 3-point Likert scale to provide a qualitative measure of recommendation relevance. In the actual application test, I can see that for the model established in this paper, its overall AUC is 0.843, which is significantly higher than the basic model, and also higher than the PNN and DIN of 0.829 and 0.831. On the basis of this, I have carried out the calculation of RelaImpr indexes, and the results show that the DP-GCN method has a more obvious effect of improving the performance of the DP-GCN method, which is more than 10%. 10%. In order to better test the effect of the actual application, I selected the online shopping users in our region to judge and analyze the satisfaction of the recommendationrecommendation results of different models, and its overall results are shown in Fig 8:

In Fig 8, for the recommendation satisfaction analysis of different users under different models, the overall satisfaction level of the DP-GCN method proposed in this paper is higher than that of other pairs of methods, while in the view of the method that does not use reinforcement learning, its overall satisfaction level is at a lower level. Due to the small number of users tested and the overall number of target goods recommendation, there may be some bias, but from the perspective of average satisfaction, the average satisfaction level of the DP-GCN method exceeds 80%, which proves that the method has a good application effect. The high satisfaction index indicates the effectiveness of the DP-GCN method in understanding and predicting user needs. In the field of personalized marketing, being able to accurately identify users’ preferences and provide product recommendationes that meet their expectations is a core task.DP-GCN makes recommendationes more accurate by using graph neural networks to deeply mine the complex relationship between users and products, thus improving user satisfaction. This is extremely valuable for organizations because high user satisfaction is usually directly related to higher user retention and better conversion rates, which directly affects the revenue and market share of the organization. Although the study mentions that there may be bias due to the limited number of test users and recommendationed products, the result of an average satisfaction rate of more than 80% is still persuasive, which indicates that the DP-GCN method has high reliability and generalizability in practical applications.

## 5. Discussion

In this paper, a user interest recommendation framework DP-GCN based on deep learning and reinforcement learning methods is proposed for the needs of user behavior prediction and analysis in the process of e-commerce marketing.The framework utilizes graph neural network (GCN) for in-depth analysis and feature extraction to deal with the three-object heterogeneous network that covers users, products and queries, which significantly improves the accuracy and effect of personalized recommendation.

Among the traditional recommender system approaches, such as PNN (Product-based Neural Network) and DIN (Deep Interest Network), although they are able to handle certain nonlinear relationships and feature interactions, they often show limitations in dealing with a wide range of heterogeneous relationships and dynamic user behavior patterns. In contrast, the DP-GCN framework is able to capture and utilize more comprehensively the complex interactions between users and goods, and between queries and these elements, by constructing a graph structure that contains multiple entity and relationship types. In addition, the application of GCN allows each node to be analyzed not just independently, but in a global graph structure that considers the influence of its neighbors, thus enabling more in-depth feature fusion.One of the key innovations in the DP-GCN framework is the introduction of a dynamic feature fusion and decision-making process based on the DDPG strategy. This strategy allows the model to dynamically adjust its recommendation strategy in a real-time environment, optimizing its behavior by continuously learning from environmental changes and user feedback. This not only enhances the model’s adaptability to the current data state, but also provides the model with the ability to predict future trends, which greatly improves the flexibility and effectiveness of the recommender system. In addition, DP-GCN makes the recommendation results more accurate and personalized by combining static and dynamic features, and adding basic user information and historical behavioral features to the feature fusion layer. This combination uses a multi-layer neural network layer to further process these features, ensuring that a variety of information is fully utilized and improving the predictive ability of the model. In terms of performance evaluation, the DP-GCN framework demonstrates higher AUC performance than traditional methods such as PNN and DIN on multiple public datasets, which proves its effectiveness and superiority in real-world applications. This performance enhancement stems from the framework’s in-depth mining of complex data relationships and its high adaptability to environmental dynamics. My model performs well in scalability and can handle larger and more diverse datasets. Firstly, the local information aggregation feature of GCN ensures that as the graph size increases, only relevant nodes and their neighbors are computed, avoiding redundant calculations and improving efficiency. Secondly, by integrating the DDPG algorithm, the model can dynamically adjust recommendation strategies when user behavior changes, without the need for frequent global retraining, ensuring the accuracy of real-time personalized recommendations. In addition, the model supports small batch training and parallel processing, which can divide large datasets into subsets or subgraphs for independent computation, thereby improving processing speed. Meanwhile, the compatibility of the framework with heterogeneous networks enables it to effectively integrate multiple data sources and maintain stable performance even when data becomes more complex and diverse. Therefore, DP-GCN not only provides an important technological advancement for current recommender system technologies, but also provides valuable experience and methodological guidance for the future development of personalized recommendation and decision support systems in complex and dynamic environments [27]. At the same time, in the future application process, the model strictly complies with regulations such as GDPR and CCPA to ensure user privacy and data security. Firstly, the model adopts a data minimization strategy, collecting only necessary information related to personalized recommendations and using anonymization and de identification processing to reduce the risk of personal information leakage. Secondly, I focus on user control and transparency, ensuring that users can access, delete, or correct their data, and choose to opt out of personalized recommendation services at any time, in compliance with legal requirements for user data control [28]. In terms of data security, I apply encryption and access control technologies to ensure the security of data during transmission and storage. At the same time, by establishing compliance management and audit mechanisms, we regularly inspect data usage to prevent violations and data abuse. These measures ensure that the model not only achieves efficient personalized marketing, but also complies with privacy protection regulations, providing users with a reliable and secure personalized service experience.

The DP-GCN framework provides significant value in personalized marketing and sustainable market segmentation targeting by enabling more precise and efficient marketing strategies through in-depth analysis and fusion of complex interactions of users, goods and queries. This graph neural network-based approach identifies subtle patterns and trends across large data sets, helping companies not only optimize for existing markets, but also discover potential market segments that can drive sustainable market growth and innovation. When applying the DP-GCN framework, enterprises are able to adjust the recommendation system in real time according to dynamic changes in user behavior and preferences, providing product recommendationes that are more in line with users’ current needs. The flexibility and adaptability of this strategy is crucial to responding to rapidly changing market environments, especially nowadays when consumer preferences are diversified and personalized experiences are pursued. However, in practical application, attention needs to be paid to the quality of data and the meticulousness of processing. The comprehensiveness, accuracy and timeliness of the data directly affect the effectiveness of the model, so a strict data management and updating mechanism needs to be established [29]. At the same time, the transparency and interpretability of the model is also key, especially when dealing with user data and privacy, to ensure compliance with relevant laws, regulations and ethical standards, and to build user trust is a prerequisite for the implementation of such advanced technologies. While this study demonstrates the effectiveness of personalized marketing through behavioral data, it is important to acknowledge the ethical and regulatory considerations inherent in such systems. Real-world deployment must address user data privacy and adhere to data protection regulations such as the General Data Protection Regulation. Furthermore, personalized systems carry the risk of reinforcing existing user biases or unintentionally excluding certain user groups. Future work should incorporate privacy-preserving techniques, ensure transparency in data usage, and adopt fairness-aware algorithms to mitigate bias and promote equitable personalization outcomes [30]. Finally, continuous technical updates and team training are also necessary to ensure the long-term effective operation of the DP-GCN framework.

## 6. Conclusion

The DP-GCN framework based on graph neural networks proposed in this study provides an effective way to solve the problems of user behavior prediction and commodity recommendation in personalized marketing. By combining deep learning and reinforcement learning techniques, a recommendation system capable of processing and analyzing large-scale heterogeneous information networks is constructed, and deep mining and dynamic feature fusion of the complex relationships among users, commodities and queries are successfully realized, thus significantly improving the prediction accuracy of the model and the recommendation effect. The experimental results verify the superior performance of the framework under public datasets, and its AUC performance is significantly better than traditional personalized recommendation methods, such as PNN and DIN. In the analysis of the self-built real dataset, DP-GCN improves its RelaImpr by more than 10% compared to the base model. This research result not only promotes the further development of personalized marketing technology, but also provides new technical means and strategy support for enterprises to achieve more accurate market segmentation and efficient user service.

In future research, I will further extend the application scope of the framework to cross-domain information recommendation by integrating data from multiple markets and diverse consumer behaviors. This will allow us to explore how preferences and behaviors transfer across different domains, enhancing the framework’s effectiveness in personalized marketing. Additionally, I plan to incorporate advanced AI techniques, such as meta-learning and adaptive algorithms, to improve the model’s ability to generalize across domains and adapt to new contexts with minimal retraining. Through continuous optimization, we aim to provide more robust technical support for cross-domain marketing strategies and personalized user experiences. Ultimately, this will contribute to advancing marketing technology by addressing the challenges of fragmented user behavior across multiple fields and promoting sustainable innovation in the industry.

## References

[1] Stachl C, Au Q, Schoedel R, Gosling SD, Harari GM, Buschek D, et al. Predicting personality from patterns of behavior collected with smartphones. Proc Natl Acad Sci U S A. 2020;117(30):17680–7. doi: 10.1073/pnas.1920484117 32665436 PMC7395458

[2] Qi L, Hu C, Zhang X, Khosravi MR, Sharma S, Pang S, et al. Privacy-Aware Data Fusion and Prediction With Spatial-Temporal Context for Smart City Industrial Environment. IEEE Trans Ind Inf. 2021;17(6):4159–67. doi: 10.1109/tii.2020.3012157

[3] G. Martín A, Fernández-Isabel A, Martín de Diego I, Beltrán M. A survey for user behavior analysis based on machine learning techniques: current models and applications. Appl Intell. 2021;51(8):6029–55. doi: 10.1007/s10489-020-02160-x

[4] Xu Z, Zhu G, Metawa N, Zhou Q. Machine learning based customer meta-combination brand equity analysis for marketing behavior evaluation. Information Processing Manag. 2022;59(1):102800. doi: 10.1016/j.ipm.2021.102800

[5] Hagen L, Uetake K, Yang N, Bollinger B, Chaney AJB, Dzyabura D, et al. How can machine learning aid behavioral marketing research?. Mark Lett. 2020;31(4):361–70. doi: 10.1007/s11002-020-09535-7

[6] Huang AYQ, Lu OHT, Yang SJH. Effects of artificial Intelligence–Enabled personalized recommendations on learners’ learning engagement, motivation, and outcomes in a flipped classroom. Comp Education. 2023;194:104684. doi: 10.1016/j.compedu.2022.104684

[7] Du Z, Zhang T, Chen Y, Ai L, Wang X. A content and user-oblivious video-recommendation algorithm. Simulation Modelling Practice and Theory. 2011;19(9):1895–912. doi: 10.1016/j.simpat.2011.04.012

[8] Zheng N, Li Q. A recommender system based on tag and time information for social tagging systems. Expert Systems with Applications. 2011;38(4):4575–87. doi: 10.1016/j.eswa.2010.09.131

[9] Bobadilla J, Serradilla F, Bernal J. A new collaborative filtering metric that improves the behavior of recommender systems. Knowledge-Based Systems. 2010;23(6):520–8. doi: 10.1016/j.knosys.2010.03.009

[10] Zhou G, Zhu X, Song C. Deep interest network for click-through rate prediction. In: Proceedings of the 24th ACM SIGKDD international conference on knowledge discovery & data mining. 2018. 1059–68.

[11] Zhang W, Du T, Wang J. Deep learning over multi-field categorical data: –a case study on user response prediction. In: Advances in Information Retrieval: 38th European Conference on IR Research, ECIR 2016, Padua, Italy, March 20–23, 2016. Proceedings. Padua, Italy; 2016. 45–57.

[12] Wang H, Wang N, Yeung DY. Collaborative deep learning for recommender systems. In: KDD 2015, 2015.

[13] Yao W, Dubois C, Zheng AX. Collaborative denoising auto-encoders for top-n recommender systems. In: the Ninth ACM International Conference. 2016.

[14] Oord AVD, Dieleman S, Schrauwen B. Deep content-based music recommendation. Adv Neural Information Processing Systems. 2013.

[15] Zheng L, Noroozi V, Yu PS. Joint deep modeling of users and items using reviews for recommendation. In: the Tenth ACM International Conference, 2017.

[16] Hidasi B, Karatzoglou A, Baltrunas L. Session-based recommendations with recurrent neural networks. Comp Sci. 2015.

[17] Liu Q, Wu S, Wang L, Tan T. Predicting the next location: A recurrent model with spatial and temporal contexts. In: Proceedings of the 30th AAAI Conference on Artificial Intelligence. 2016.

[18] Wu CY, Ahmed A, Beutel A. Recurrent recommender networks. In: Tenth ACM International Conference on Web Search & Data Mining. 2017.

[19] Abu-El-Haija S, Kapoor A, Perozzi B. N-gcn: Multi-scale graph convolution for semi-supervised node classification. In: Uncertainty in artificial intelligence. 2020. 841–51.

[20] Min Y, Wenkel F, Wolf G. Scattering GCN: Overcoming Oversmoothness in Graph Convolutional Networks. Adv Neural Inf Process Syst. 2020;33:14498–508. 37337543 PMC10277640

[21] Dong Y, Zou X. Mobile robot path planning based on improved DDPG reinforcement learning algorithm. In: 2020 IEEE 11th International Conference on Software Engineering and Service Science (ICSESS). 2020. 52–6.

[22] He N, Yang S, Li F. A-DDPG: Attention mechanism-based deep reinforcement learning for NFV. In: 2021 IEEE/ACM 29th International Symposium on Quality of Service (IWQOS). 2021. 1–10.

[23] Barbado R, Araque O, Iglesias CA. A framework for fake review detection in online consumer electronics retailers. Information Processing Management. 2019;56(4):1234–44. doi: 10.1016/j.ipm.2019.03.002

[24] Zhang Y, Chen L, Yang S, et al. PICASSO: Unleashing the Potential of GPU-centric Training for Wide-and-deep Recommender Systems[C]//2022 IEEE 38th International Conference on Data Engineering (ICDE). IEEE, 2022: 3453–66.

[25] Sandri S, Molinari A. Preference learning in food recommendation: the “myfood” case study. In: 2023 3rd International Conference on Electrical, Computer, Communications and Mechatronics Engineering (ICECCME). 2023. 1–6.

[26] Song S, Zhang T, Huang Y, Zhang B, Guo Y, He Y, et al. Urinary metabolites of neonicotinoid insecticides: levels and recommendations for future biomonitoring studies in China. Environ Sci Technol. 2020;54(13):8210–20. doi: 10.1021/acs.est.0c01227 32388996

[27] Shen T, Zhang Y, Wang J, Zhang X. Graphs get personal: learning representation with contextual pretraining for collaborative filtering. Appl Intell. 2023;53(24):30416–30. doi: 10.1007/s10489-023-05144-9

[28] Zheng Y, Wei P, Chen Z. Graph-convolved factorization machines for personalized recommendation. IEEE Transactions on Knowledge and Data Engineering. 2021;35(2):1567–80.

[29] Meng X, Wang S, Shu K, Li J, Chen B, Liu H, et al. Personalized privacy-preserving social recommendation. AAAI. 2018;32(1). doi: 10.1609/aaai.v32i1.11714

[30] Javeed D, Saeed MS, Kumar P, Jolfaei A, Islam S, Islam AKMN. Federated learning-based personalized recommendation systems: an overview on security and privacy challenges. IEEE Trans Consumer Electron. 2024;70(1):2618–27. doi: 10.1109/tce.2023.3318754

